# Materia Medica

**Published:** 1878-04

**Authors:** 


### Books Reviewed.

*Materia Medica, for the use of Students.* By JOHN B. BIDDLE, M. D. Eighth Edition. Philadelphia: Lindsay and Blakiston. 1878. Buffalo: T. H. Butler. pp. 462. Price, $4.00.

No better evidence of the opinion in which this volume is held, could be given, than the fact that the seventh edition, has been exhausted in little more than a year. It has been carefully revised, much of it has been recast and even rewritten and many new articles have been added. An earlier edition was noticed in a previous number of this Journal and we fully agree in the high estimate placed upon it in that article. We know of no work upon Materia Medica which excells it or is of more value to the student and busy practitioner. Any physician unprovided should immediately purchase it.

